# Peroxiredoxin II negatively regulates BMP2-induced osteoblast differentiation and bone formation via PP2A Cα-mediated Smad1/5/9 dephosphorylation

**DOI:** 10.1038/s12276-019-0263-x

**Published:** 2019-06-03

**Authors:** Kyeong-Min Kim, Do-Young Kim, Dong-Seok Lee, Jung-Woo Kim, Jeong-Tae Koh, Eun-Jung Kim, Won-Gu Jang

**Affiliations:** 1 Department of Biotechnology, School of Engineering, Daegu University, Gyeongbuk, 38453 Republic of Korea; 2 Research Institute of Anti-Aging, Daegu University, Gyeongbuk, 38453 Republic of Korea; 3 College of Natural Sciences, Kyungpook National University, Daegu, Republic of Korea; 4 Department of Pharmacology and Dental Therapeutics, School of Dentistry, Chonnam National University, Gwangju, 61186 Republic of Korea; 5 Department of Immunology, Kyungpook National University School of Medicine, Daegu, 41944 Republic of Korea

**Keywords:** Cell signalling, Mesenchymal stem cells

## Abstract

Peroxiredoxin II (Prx II), an antioxidant enzyme in the Prx family, reduces oxidative stress by decreasing the intracellular ROS levels. Osteoblast differentiation is promoted by bone morphogenetic protein 2 (BMP2), which upregulates the expression of osteoblast differentiation marker genes, through Smad1/5/9 phosphorylation. We found that Prx II expression was increased by a high dose of lipopolysaccharide (LPS) but was not increased by a low dose of LPS. Prx II itself caused a decrease in the osteogenic gene expression, alkaline phosphatase (ALP) activity, and Smad1/5/9 phosphorylation induced by BMP2. In addition, BMP2-induced osteogenic gene expression and ALP activity were higher in Prx II knockout (KO) cells than they were in wild-type (WT) cells. These inhibitory effects were mediated by protein phosphatase 2A Cα (PP2A Cα), which was increased and is known to induce the dephosphorylation of Smad1/5/9. The overexpression of Prx II increased the expression of PP2A Cα, and PP2A Cα was not expressed in Prx II KO cells. Moreover, PP2A Cα reduced the level of BMP2-induced osteogenic gene expression and Smad1/5/9 phosphorylation. LPS inhibited BMP2-induced Smad1/5/9 phosphorylation and the suppressed phosphorylation was restored by the PP2A inhibitor okadaic acid (OA). Bone phenotype analyses using microcomputed tomography (μCT) revealed that the Prx II KO mice had higher levels of bone mass than the levels of the WT mice. We hypothesize that Prx II has a negative role in osteoblast differentiation through the PP2A-dependent dephosphorylation of Smad1/5/9.

## Bone health: Enzyme protects against damage during stress

An antioxidant enzyme actively works to reduce bone synthesis under oxidative stress conditions in order to protect bone cells from damage and cell death. Bone is generated by cells called osteoblasts, which differentiate from stem cells. In osteoporosis and diabetes, excessive reactive oxygen species (ROS) within cells can disrupt osteoblast differentiation. South Korean researchers led by Eun-jung Kim at Kyungpook National University, Daegu, and Won-Gu Jang at Daegu University, Gyeongbuk, have shown that an antioxidant enzyme, peroxiredoxin II (PrxII), helps regulate bone formation under oxidative stress. The team generated PrxII-deficient mice and compared them with healthy normal mice. Under oxidative stress conditions, the mice had higher bone mass and higher expression of genes related to bone formation than the normal mice. PrxII limits osteoblast differentiation during elevated ROS by influencing associated protein activity and signalling pathways.

## Introduction

Peroxiredoxins (Prxs) are antioxidant enzymes with six subtypes (Prx I–VI) and they mainly reduce the reactive oxygen species (ROS) levels^1^. These subtypes have different locations within the cell; Prx I, II, and VI are present in the cytoplasm, Prx III plays a role in the mitochondria, Prx IV plays a role in the Golgi apparatus, and Prx V performs an antioxidant function in peroxisomes^2^. Prx II protects cells from oxidative stress by reducing the levels of ROS^3,4^. The levels of ROS, which are produced during cellular respiration, are regulated to reduce oxidative damage^5^. Intracellular ROS is an important regulator of cell proliferation and differentiation in various cells, including stem cells and cancer cells^6^. In addition, ROS plays a role in the regulation of gene expression as a secondary signal transducer in a variety of cell and biological processes, such as the cytokine response, growth factor and hormone treatment response, ion transport, transcription, neuromodulation, and apoptosis^7–9^. Oxidative stress occurs by the excessive accumulation of ROS and has been implicated in various metabolic diseases, such as diabetes and osteoporosis^10–14^.

The bone is a dynamic tissue; osteoblast-induced bone formation and osteoclast-induced bone loss occur throughout the lifetime^15^. Osteoblasts differentiate from mesenchymal stem cells, and they are regulated by hormones and cytokines^16,17^. BMP2 is one of the most important cytokines in bone repair, bone formation, and osteoblast differentiation^18,19^. BMP2-induced osteoblast differentiation regulates the transcription of osteogenic genes, such as the DNA-binding protein inhibitor (Id1), distal-less homeobox 5 (Dlx5), and runt-related transcription factor 2 (Runx2) through the phosphorylation of Smad1/5/9^20,21^. These genes also upregulate the expression of next-stage markers, including ALP and OC^20,22,23^.

Protein phosphatases modulate various important cellular processes, such as protein synthesis, the cell cycle, and glycogen metabolism^24^. PP2A is a large family of heterotrimeric phosphatases in eukaryotic cells that regulate numerous signaling pathways, such as those involved in metabolism, the kinase cascade, cell growth, and cell death^25,26^.

Prx I and Prx V in osteoblasts have been studied^27,28^, but the role of Prx II in osteoblast differentiation has not yet been elucidated. In this study, we demonstrate that Prx II causes a decrease in BMP2-induced osteoblast differentiation through the upregulation of PP2A Cα expression, which inhibits the phosphorylation of Smad1/5/9.

## Materials and methods

### Reagents and antibodies

Lipopolysaccharide (LPS) was purchased from Sigma-Aldrich (St. Louis, MO). Dulbecco’s Modified Eagle Medium (DMEM), phosphate-buffered saline, penicillin–streptomycin, and 0.25% trypsin-EDTA were obtained from GIBCO-BRL (Grand Island, NY). Fetal bovine serum (FBS) was purchased from MP Biomedicals (Seoul, Korea). Emerald Amp GRPCR Master Mix was purchased from TaKaRa (Shiga, Japan), and AmpiGene^TM^ qPCR Green Mix Hi-ROX was purchased from Enzo (Farmingdale, NY). Recombinant human BMP2 was purchased from Cowellmedi Co. (Busan, Korea). Antibodies against Prx II and β-actin were obtained from Santa Cruz Biotechnology (Dallas, TX). Antibodies against PP2A Cα, Smad, and phospho-Smad (p-Smad) were purchased from Cell Signaling Technology (Cambridge, MA).

### Cell culture

The mouse embryonic mesenchymal stem cell line C3H10T1/2 (ATCC, Manassas, VA) was maintained in DMEM containing 10% FBS, 100 units/mL penicillin, and 100 μg/ml streptomycin in humidified air with 5% CO_2_ at 37 °C. Osteoblast differentiation was induced by adding 0.25 μg/ml rhBMP2. The culture medium was replaced every 2 days. To induce ROS, cells were cultured in a medium with LPS (0.05 and 1 μg/ml) and H_2_O_2_ (5 and 100 μM) for 30 min.

### Animals

Wild-type (WT) and Prx II-knockout (Prx II KO) male mice (6-weeks old) with a C57BL/6 background were maintained in accordance with the institutional guidelines of the Committee for Laboratory Animal Care and Use of Daegu University. Animals were maintained under standard environmental conditions (temperature at 20–22 °C, humidity at 50–60%, and 12-h dark/light cycles) with free access to food and water.

### RT-PCR and real-time PCR analyses

Total RNA was isolated from cells using TRIzol Reagent (Bio Science Technology, Daegu, Korea) according to the manufacturer’s instructions. Reverse transcription was performed using 3 μg of total RNA as the template. RT-PCR conditions were as follows: initial denaturation at 95 °C for 5 min; this was followed by a three-step cycle of denaturation at 95 °C for 30 s, annealing at the optimal temperature of each primer pair for 30 s, and extension at 72 °C for 30 s; a final extension was performed at 72 °C for 5 min. Real-time PCR was performed using 3 μg of total RNA as the template. The PCR conditions were as follows: initial denaturation at 95 °C for 5 min, 45 cycles of denaturation at 95 °C for 30 s, annealing at the optimal temperature of each primer pair for 30 s, and extension at 72 °C for 30 s. The final extension was performed at 72 °C for 5 min. The expression levels were normalized to those of endogenous β-actin, and the data were analyzed using the 2^ΔΔ–C^_T_ method^29^. The primer sequences of RT-PCR were as follows: mouse β-actin forward, 5′-TTC TTT GCA GCT CCT TCG TTG CCG-3′, and reverse, 5′- TGG ATG GCT ACG TAC ATG GCT GGG-3′; mouse Id1 forward, 5′-ATG AAG GTC GCC AGT GGC AGT-3′, and reverse, 5′- ACT TTG CGG TTC TGG GGC AGG-3′; mouse Dlx5 forward, 5′-CAG AAG AGT CCC AAG CAT CC-3′, and reverse, 5′-GAG CGC TTT GCC ATA AGA AG-3′; mouse Runx2 forward, 5′-AGA GTC AGA TTA CAG ATC CCA GG -3′, and reverse, 5′-TGG CTC TTC TTA CTG AGA GAG G-3′; mouse Prx II forward, 5′-AGG ACT TCC GAA AGC TAG GC-3′, and reverse, 5′- TTG ACT GTG ATC TGG CGA AG-3′. The primer sequences of qPCR were as follows: mouse β-actin forward, 5′-TTC TAC AAT GAG CTG CGT GTG-3′, and reverse, 5′-GGG GTG TTG AAG GTC TCA AA-3′; mouse Id1 forward, 5′-CTT CAG GAG GCA AGA GGA AA-3′, and reverse, 5′-CAA ACC CTC TAC CCA CTG GA-3′; mouse Dlx5 forward, 5′-GCC CAC CAA CCA GCC AGA GA-3′, and reverse, 5′-GCG AGG TAC TGA GTC TTC TGA AAC C-3′; mouse PP2A Cα forward, 5′-CAC CAT CCA TAG ACA CAC TG-3′, and reverse, 5′-GCA CCA GTT ATA TCC CTC CA-3′; mouse Prx II forward, 5′-AGG ACT TCC GAA AGC TAG GC-3′, and reverse, 5′-TTG ACT GTG ATC TGG CGA AG-3′.

### Measurement of intracellular ROS

To measure the intracellular ROS, a total ROS Detection Kit (Enzo Scientific, Farmingdale, NY) was used according to the instructions provided by the manufacturer. C3H10T1/2 cells were treated with 1 and 0.05 μg/ml LPS for 30 min. Images were acquired using a fluorescence microscope.

### ALP staining

C3H10T1/2 and Prx II KO cells were cultured with rhBMP2 (0.25 μg/mL) for 4 days. Staining was performed using standard protocols. Briefly, the cultured cells were fixed with 10% formaldehyde, rinsed twice with deionized water, and treated with BCIP^®^/NBT Solution (Sigma-Aldrich) for 15 min. After additional washing, the stained cultures were imaged.

### Western blot analysis

The total cells were harvested and lysed using an EzRIPA Lysis Kit (ATTO Technology, Tokyo, Japan) and were then centrifuged at 12,000×*g* for 10 min at 4 °C. The total proteins were quantified using the Bradford assay, separated by SDS-PAGE, and then transferred onto a PVDF membrane. After blocking in 5% skimmed milk prepared in Tris-buffered saline containing Tween 20, the membrane was incubated with specific primary antibodies (1:1000). Signals were detected using an ECL reagent (Advansta, Menlo Park, CA). Densitometric analysis of the blotted membrane was performed using a FUSION solo analyzer system (Vilber Lourmat, Eberhardzell, Germany).

### CRISPR/Cas9 plasmid for Prx II

A Cas9-expression plasmid was purchased from Addgene (Cambridge, MA). The sgRNA plasmid was constructed by subcloning the crRNA and tracrRNA sequences under the control of the hU6 promoter from the pCLIIP-ALL-EFS-Puro cloning vector (TransOMIC Technologies, Huntsville, AL) into the minimal PUC18 backbone plasmid. To knock out the Prx II gene, oligonucleotides containing target sequences for exon 1 were synthesized (Bioneer, Daejeon, Korea) and inserted into the sgRNA plasmid that had been digested with BsmBI.

### Transfection and the T7E1 assay

Cells were transfected with the plasmids encoding Cas9 and the sgRNA using the 4D-Nucleofector System (Amaxa, Koeln, Germany) at a molecular weight ratio of 1:2 (plasmid encoding Cas9:plasmid encoding sgRNA). Seven hundred cells were spread over a 100-mm culture dish to form single-cell-derived colonies. Then, 5–10 cells from a colony were collected and lysed. Mutant colonies were selected via PCR and the T7E1 assay. To amplify the Prx II gene including the exon 1 region, nested PCR was performed using sequence-specific primers (forward, 5′-GAATATGAGCGCTCCTTCCA-3′; reverse (1), 5′-GAGAACTGAGACTCCTTTGG-3′; and reverse (2), 5′-GGGAAGTCAGTGCTAACTTC-3′). PCR products were denatured at 95 °C and were reannealed by reducing the temperature to randomly generate heteroduplex DNA. The heteroduplex DNA was then treated with 5 units of T7 endonuclease 1 (New England Biolabs, Beverly, MA) for 1 h at 37 °C and was analyzed by electrophoresis on a 2% agarose gel.

### Microcomputed tomography (μ-CT) scanning

The femora and tibiae of 6-week-old wild-type (WT) and Prx II KO mice were obtained and fixed in 4% paraformaldehyde at 4 °C overnight. The microarchitecture of the femora and tibiae was examined using a microcomputed tomography apparatus (Skyscan1172; Bruker, Kontich, Belgium). Scan conditions were performed as described^30^. To measure trabecular bone parameters, 1-mm slice images were taken immediately adjacent to the distal growth plate. To measure the cortical bone parameters, we analyzed the 1-mm slices from the middle of the bone using the CT-Analyzer software (Skyscan) and the three-dimensional model visualization software CTvol (Skyscan).

### Statistical analysis

All experiments were performed at least three times. Statistical analysis was performed using Student’s *t* test or analysis of variance, followed by Duncan’s multiple comparison test. The results with *p* values < 0.05 were considered significant.

## Results

### The inhibition of osteoblast differentiation by LPS and the expression level of Prx II depend on the concentration

**Fig. 1:**
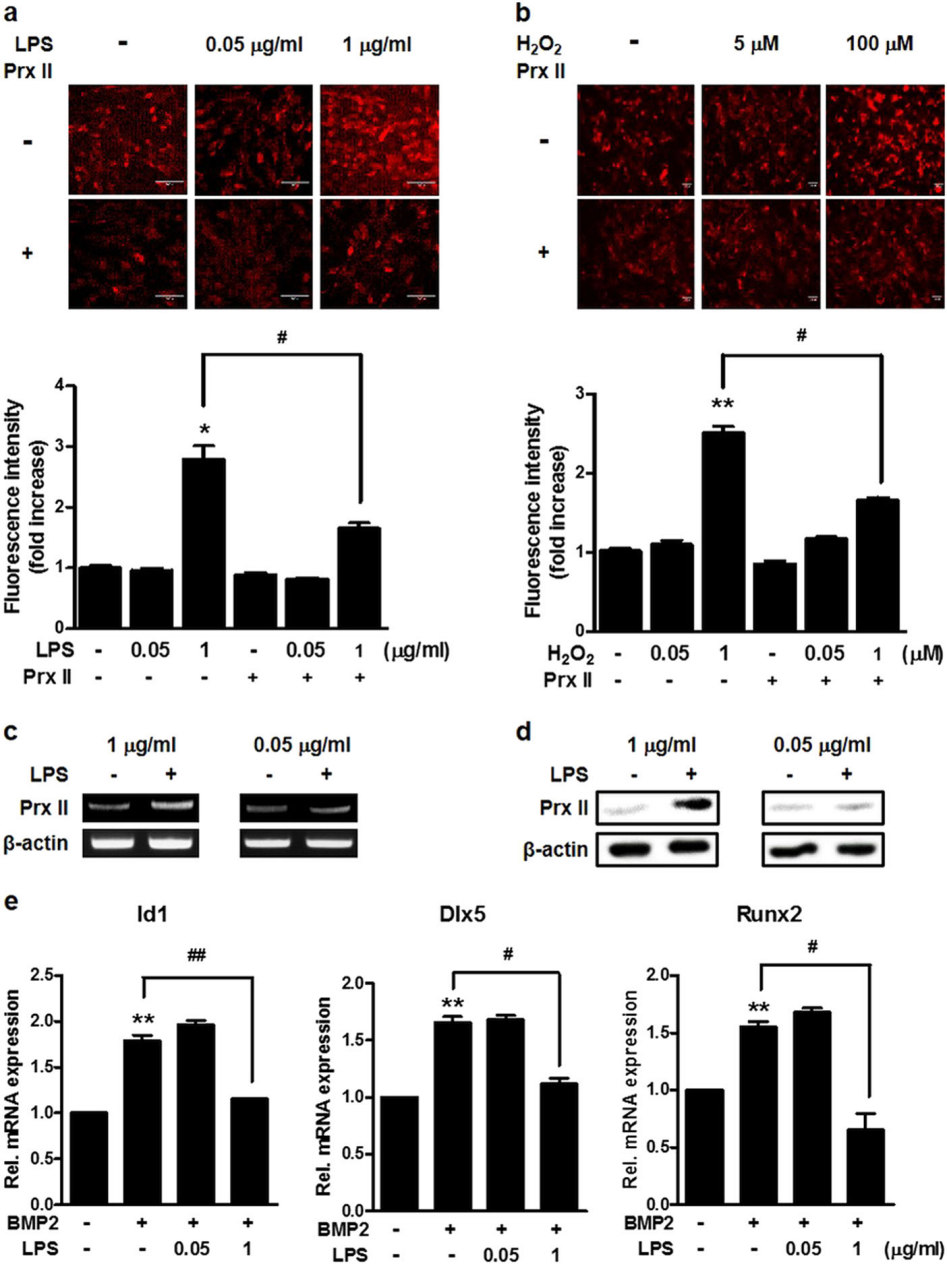
Effect of LPS on the concentration of osteoblast differentiation and Prx II expression. **a** Cells were treated with 0.05 or 1 μg/ml LPS for 30 min and DCF-DA staining was performed. Scale bar, 100 μm. The staining image is shown in the upper panel. The relative fluorescence intensity is shown in the lower panel. *p *<* 0.05 compared with the untreated control. ^#^*p* *<* 0.05 compared with the indicated group. **b** Cells were treated with 5 or 100 μM H_2_O_2_ for 30 min and DCF-DA staining was performed. Scale bar, 100 μm. The staining image is shown in the upper panel. The relative fluorescence intensity is shown in the lower panel. **p *<* 0.01 compared with the untreated control. ^#^*p* *<* 0.05 compared with the indicated group. **c** RT-PCR was performed using the total RNA isolated from cells treated with 1 or 0.05 μg/ml LPS for 1 day as the template. **d** Cells were treated with 1 or 0.05 μg/ml LPS for 1 day and Western blot assays were performed. **e** Real-time PCR was performed using total RNA isolated from C3H10T1/2 cells treated with 0.25 μg/ml BMP2, 0.05, and/or 1 μg/ml LPS for 2 days as the template. **p *<* 0.01 compared with the untreated control. ^#^*p* *<* 0.05, ^##^*p* *<* 0.01 compared with the indicated group. Data are presented as the mean ± SEM of three individual experiments

LPS is known to increase the production of ROS and to inhibit osteoblast differentiation^31^. We first examined the production of ROS by LPS in osteoblasts. As known, LPS increases the production of ROS in osteoblasts. However, low concentrations of LPS did not increase the production of ROS (Fig. 1a). Hydrogen peroxide (H_2_O_2_) treatment was similar to the LPS treatment (Fig. 1b). At this time, we found that the expression of Prx II depends on the concentration of LPS. High concentrations of LPS increased the expression of Prx II, whereas low concentrations of LPS did not increase Prx II expression (Fig. 1c). The protein levels also showed an increase in Prx II at high concentrations of LPS but not at low concentration of LPS (Fig. 1d). We examined the effect of LPS concentration on osteoblast differentiation and confirmed that the concentration of LPS inhibited osteoblast differentiation differently. For the expression of osteogenic markers, including Id1, Dlx5, and Runx2, a high concentration of LPS decreased the expression of mRNA by BMP2, but a low concentration of LPS did not (Fig. 1e). Taken together, these data indicate that the expression of the antioxidant enzyme, Prx II, is different, depending on the concentration of LPS and that LPS regulates osteoblast differentiation by BMP2 differently depending on the concentration.

### Prx II negatively regulates osteoblast differentiation

**Fig. 2:**
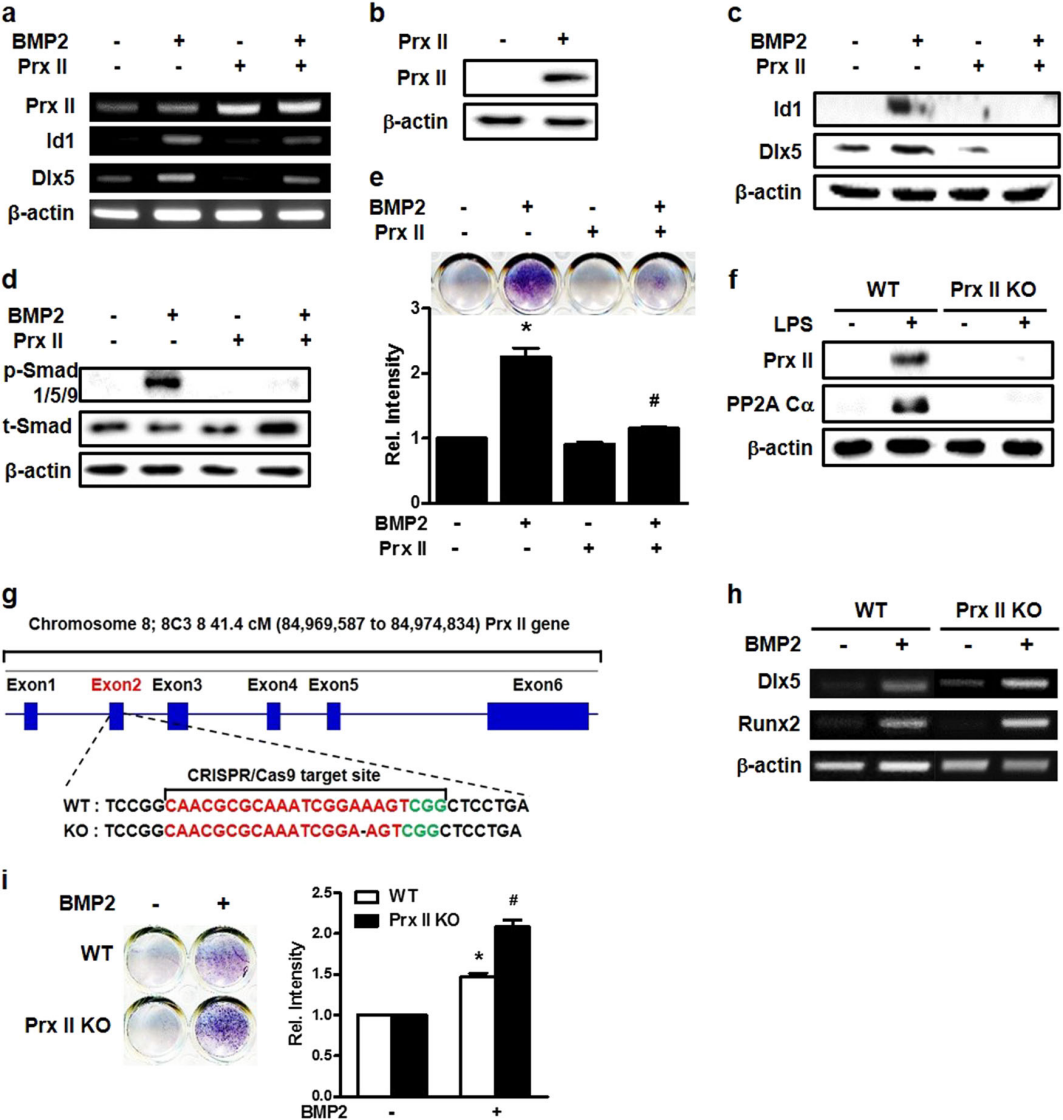
Regulation of BMP2-induced osteoblast differentiation by Prx II. **a** RT-PCR was performed using the total RNA isolated from cells that were transfected with 2 μg of the Prx II overexpression vector and that were treated with BMP2 for 2 days. Western blot analyses are shown using the indicated antibodies. **b** Cells were transfected with the Prx II overexpression vector or the control vector, and Prx II expression was measured by western blot analyses. **c** Prx II-overexpressing C3H10T1/2 cells were treated with or without BMP2 (0.25 μg/ml) for 1 day. The protein levels of Id1 and Dlx5 were measured by Western blot analyses. **d** Prx II-overexpressing C3H10T1/2 cells were treated with or without BMP2 (0.25 μg/ml) for 30 min. The protein levels of p-Smad1/5/9 and t-Smad were measured by western blot analyses. **e** Cells were transfected with the Prx II overexpression vector. Cells were treated with BMP2 for 4 days. ALP staining was performed to determine the ALP activity. The staining image is shown in the upper panel. Relative intensity is shown in the lower panel. *p *<* 0.05 as compared with the untreated control. ^#^*p* *<* 0.05 compared with the BMP-treated group. **f** Wild-type (WT) and Prx II knockout (KO) C3H10T1/2 cells were treated with 1 μg of LPS for 1 day. Western blot analyses were performed using the indicated antibodies. **g** gRNA-targeted gene locus and sequence for Prx II engineering are shown. Prx II (gRNA) was designed to target an exon of the *Prx II* gene. **h** WT and Prx II KO C3H10T1/2 cells were treated with BMP2 and RT-PCR was performed to identify osteogenic gene expression. **i** ALP staining was performed to measure ALP activity. WT and Prx II KO C3H10T1/2 cells were treated with BMP2 for 4 days. The staining image is shown in the left panel. Relative intensity is shown in the right panel. *p *<* 0.05 compared with the untreated control. ^#^*p* *<* 0.05 compared with the BMP-treated WT

We found that osteoblast differentiation and Prx II expression were regulated differently, depending on the concentration of LPS. We investigated the role of Prx II in osteoblast differentiation by inducing the overexpression of the Prx II gene using the Prx II overexpression vector. In addition, Prx II decreased the expression of the BMP2-induced osteoblast differentiation marker genes (Fig. 2a). In the BMP signaling pathway, BMP binds to BMP receptors and increases the phosphorylation of Smad1/5/9 to promote osteoblast differentiation^32^. Because Prx II overexpression reduced the expression of the osteoblast differentiation marker genes, we confirmed the phosphorylation of Smad1/5/9. Prx II overexpression caused a reduction in the protein level of osteogenic markers, such as Id1 and Dlx5, the phosphorylation of Smad1/5/9, and the ALP activity (Fig. 2b–e). To further confirm that Prx II negatively regulates osteoblast differentiation, we constructed Prx II knockout (KO) cells using the CRISPR/Cas9 system. Western blotting was performed to confirm that Prx II was knocked out. Prx II was expressed by LPS in wild-type (WT) cells, whereas Prx II expression was not induced by LPS in Prx II KO cells (Fig. 2f, g). We then performed RT-PCR to investigate the differences in the expression of osteoblast differentiation marker genes induced by BMP2 in WT and Prx II KO cells. The expression levels of the BMP2-induced osteoblast differentiation marker genes were higher in Prx II KO cells than they were in WT cells (Fig. 2h). ALP staining also revealed that the BMP2-induced ALP activity was higher in the Prx II KO cells than the activity in the WT cells (Fig. 2i). This indicates that Prx II knockout enhances BMP-induced osteoblast differentiation and that Prx II may be a negative regulator of osteoblast differentiation.

### Prx II increases the expression of PP2A Cα and inhibits osteoblast differentiation

**Fig. 3:**
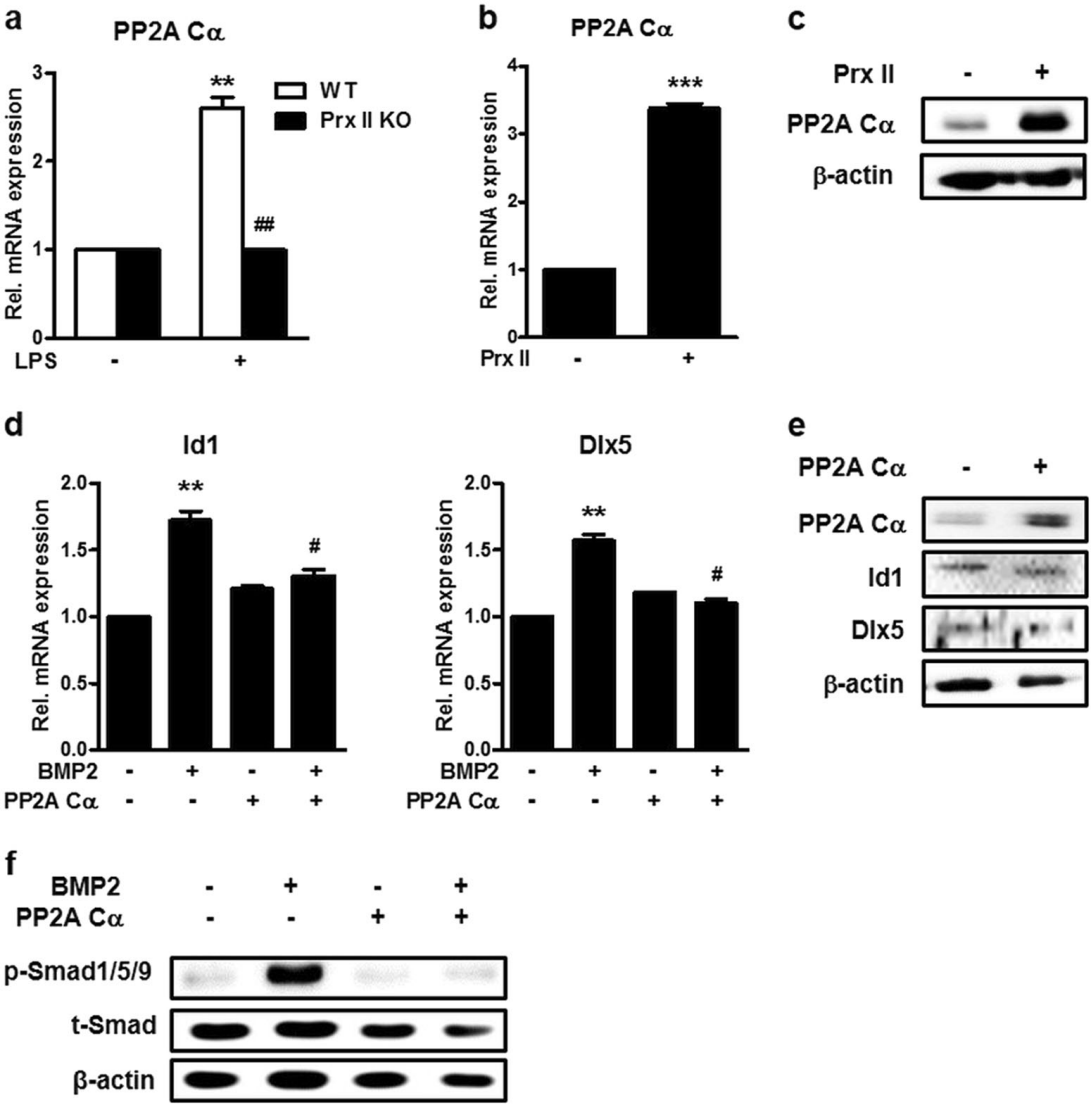
Inhibitory effect of Prx II on osteoblast differentiation by increasing PP2A Cα. **a** Wild-type and Prx II KO C3H10T1/2 cells were treated with 1 μg of LPS for 1 day. Real-time PCR was performed using the total RNA from these cells as the template. **p *<* 0.01 compared with the untreated control. ^##^*p* *<* 0.01 compared with the LPS-treated WT. **b** C3H10T1/2 cells were transfected with the Prx II overexpression vector and real-time PCR was performed using the total RNA. ***p *<* 0.005 compared with the untreated control. **c** Western blot analyses were performed using the indicated antibodies as probes. **d** C3H10T1/2 cells were transfected with the PP2A Cα overexpression vector and treated with or without BMP2 for 2 days. Real-time PCR was performed using total RNA. **p *<* 0.01 compared with untreated control. ^#^*p* *<* 0.05 compared with the BMP-treated group. The protein level of PP2A Cα, the phosphorylation of Smad1/5/9, and the total Smad are shown. **e** Cells were transfected with the PP2A Cα overexpression vector or the control vector, and PP2A Cα expression was identified by western blot analyses. **f** PP2A Cα-overexpressing C3H10T1/2 cells were treated with or without BMP2 (0.25 μg/ml) for 30 min. The protein levels of p-Smad1/5/9 and t-Smad were measured by Western blot analyses

In previous results, we showed that LPS-induced Prx II expression inhibits BMP2-induced osteoblast differentiation. To elucidate the mechanism underlying this effect, we overexpressed Prx II in WT cells. We then treated these cells and Prx II KO cells with LPS to confirm the expression of PP2A Cα. LPS caused an increase in the expression of PP2A Cα in the WT cells but did not induce the same effect in the Prx II KO cells (Fig. 3a). Prx II overexpression caused an increase in the PP2A Cα mRNA and protein levels (Fig. 3b, c). We then overexpressed PP2A Cα to examine whether PP2A Cα could regulate osteoblast differentiation. The qPCR results indicated that PP2A Cα reduced the expression of osteogenic genes (Fig. 3d). Western blot analysis showed that the overexpression of PP2A Cα did not regulate the protein levels of Id1 and Dlx5, but did cause a reduction in the phosphorylation levels of Smad1/5/9 (Fig. 3e, f). These findings suggest that PP2A Cα is involved in the mechanism underlying the inhibition of osteoblast differentiation by Prx II.

### Inhibition of PP2A Cα reverses the osteoblast differentiation inhibited by LPS

**Fig. 4:**
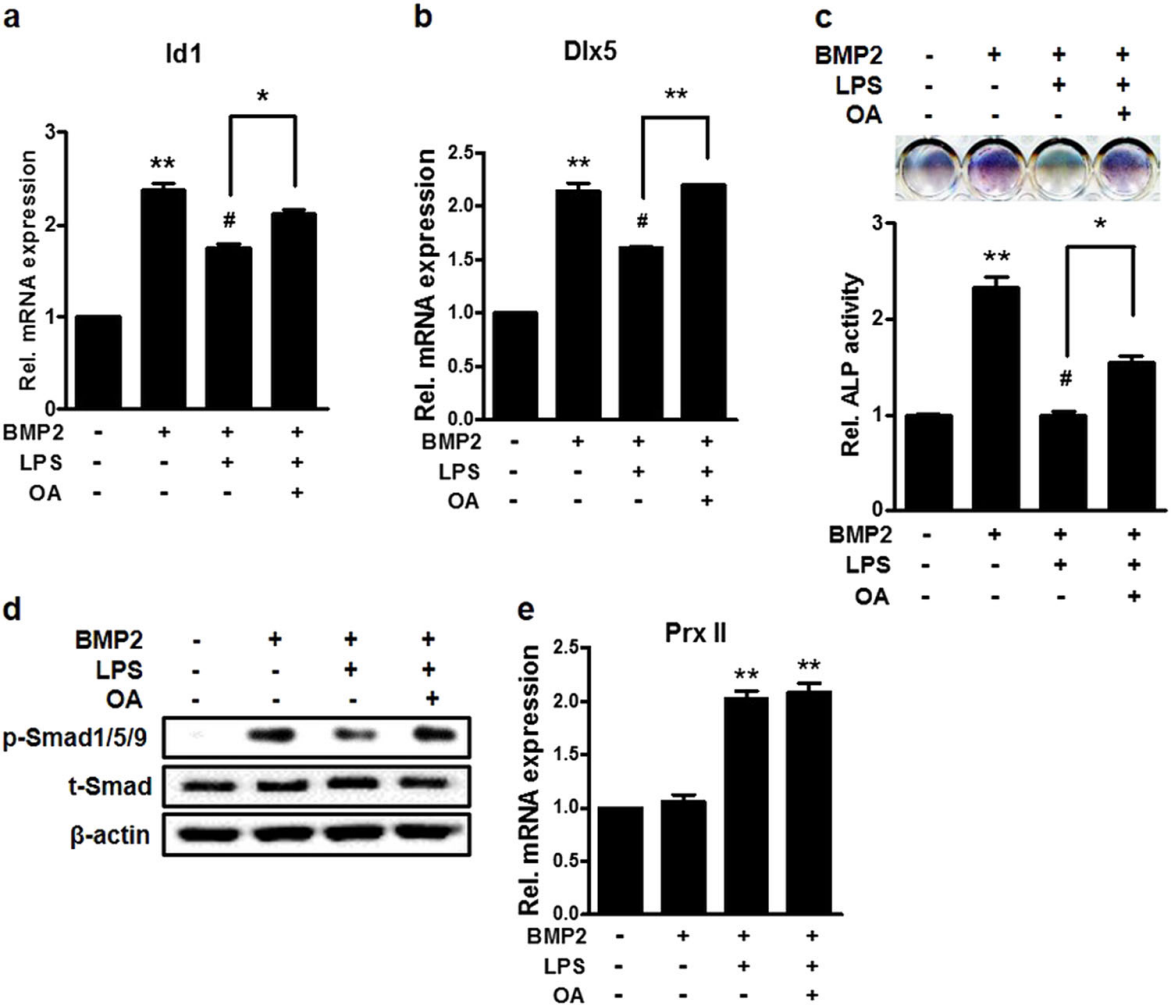
Effects of PP2A inhibition on LPS-reduced osteoblast differentiation. C3H10T1/2 cells were treated with 0.25 μg/ml BMP2, 1 μg/ml LPS, and 3 nM okadaic acid (OA) for 2 days as the template. The expression levels of **a** Id1 and **b** Dlx5 were determined using real-time PCR. **p *<* 0.01 compared with untreated control. ^#^*p* *<* 0.05 compared with the BMP2-treated group. *p *<* 0.05, **p *<* 0.01 compared with the indicated group. **c** C3H10T1/2 cells were treated with BMP2, LPS, and OA for 4 days. The upper panel represents the image of staining and the lower panel indicates the relative intensity. **p *<* 0.01 compared with untreated control. ^#^*p* *<* 0.05 compared with BMP2-treated group. *p *<* 0.05 compared with the indicated group. **d** Western blot analyses were performed using the indicated antibodies as probes. **e** Real-time PCR was performed using the total RNA isolated from C3H10T1/2 cells treated with BMP2, LPS, and OA for 2 days as the template. **p *<* 0.01 compared with the BMP2-treated group. Data are presented as the mean ± SEM of three individual experiments

To further clarify the effect of osteoblast differentiation inhibited by PP2A, we conducted an experiment using okadaic acid (OA), a PP2A Cα inhibitor. As a result of confirming the expression of Id1 and Dlx5, osteogenic genes, LPS reduced mRNA expression by BMP2 as known. However, OA treatment inhibited the mRNA expression decrease by LPS (Fig. 4a, b). ALP staining also showed similar patterns of mRNA expression. The inhibitory effect of LPS was decreased by OA (Fig. 4c). In addition, the phosphorylation level of Smad1/5/9 shows that the LPS effect was inhibited by OA similar to the previous results (Fig. 4d). At this time, the expression level of Prx II was not changed by treatment with OA (Fig. 4e). These results indicate that the inhibition of PP2A Cα reverses the inhibitory effect of osteoblast differentiation by LPS, indicating that PP2A Cα is a downstream signal of Prx II.

### Prx II deficiency increases bone formation in vivo

**Fig. 5:**
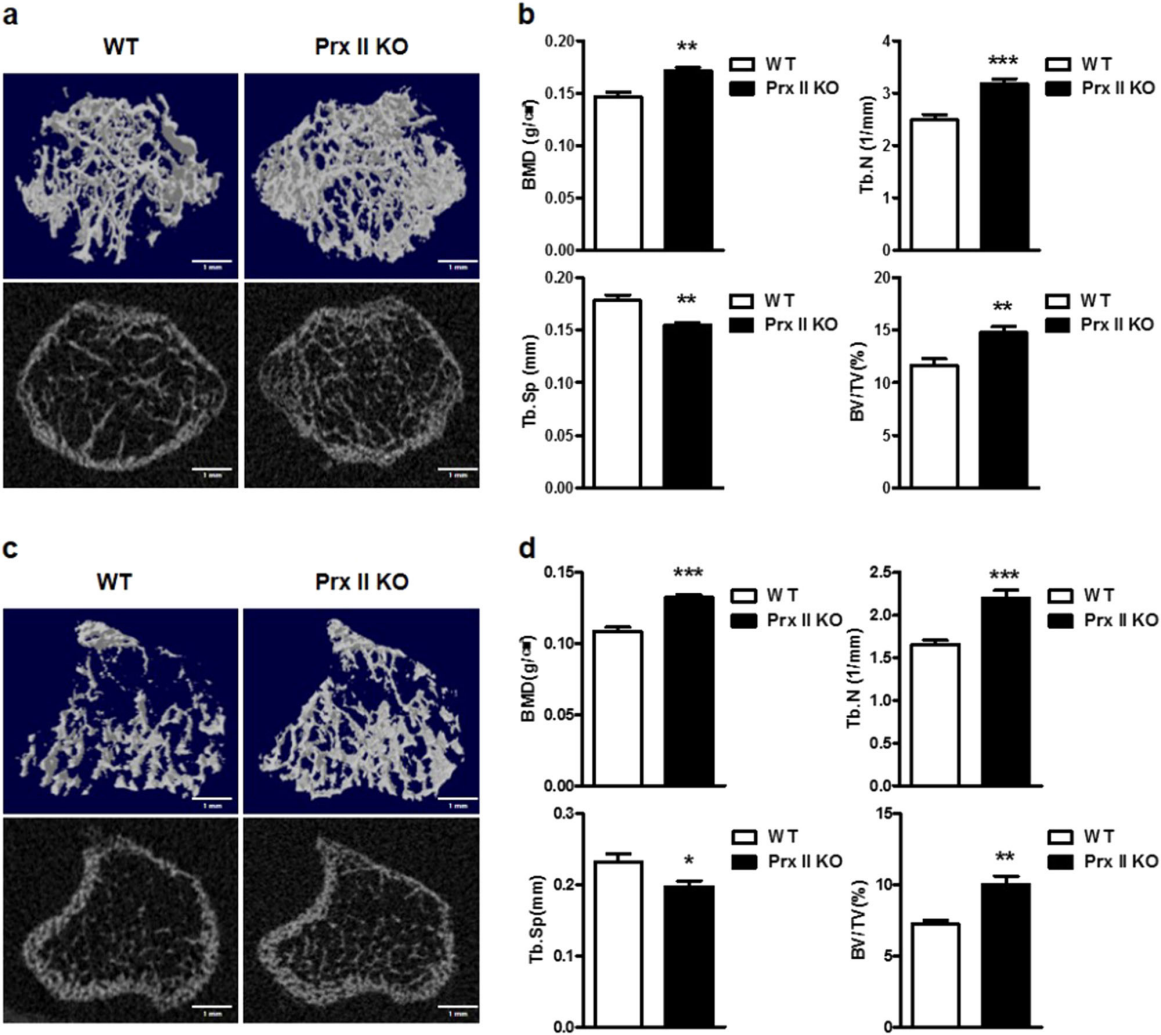
Prx II knockout results in an increase in bone parameters in vivo. μ-CT analysis of the tibiae and femora from WT and Prx II KO mice (6-week-old males, *n* = 6). **a** A 3D image of the trabecular bone of the femoral metaphysis (top) and a 2D cross-section image (bottom) are shown. Scale bar, 1 mm. **b** The parameters of the trabecular bones were quantitated and compared between the WT and Prx II KO mice. BMD, bone mineral density; Tb.N, trabecular number; BV/TV, bone volume/tissue volume ratio; Tb.Sp, trabecular separation. **c** A 3D image of the trabecular bone of tibial metaphysis (top) and 2D cross-section image (bottom) are shown. Scale bar, 1 mm. **d** The parameters of the trabecular bones were quantitated and compared between the WT and Prx II KO mice. BMD, bone mineral density; Tb.N, trabecular number; Tb.Sp, trabecular separation; BV/TV, bone volume/tissue volume ratio. *p *<* 0.05; **p *<* 0.01, and ***p *<* 0.005 compared with the WT mice

We then investigated the role of Prx II in vivo. First, we isolated femora from the WT mice and Prx II KO mice. The bone parameters of each femur were determined with μ-CT analysis (Fig. 5a). We found that the bone mineral density (BMD), trabecular number (Tb.N), and trabecular bone volume per tissue volume (BV/TV) were significantly higher in Prx II KO mice than those in WT mice. In addition, the trabecular space (Tb.Sp) was significantly lower in Prx II KO mice than that in WT mice (Fig. 5b). The μ-CT analysis of femora demonstrated that bone formation was more extensive in Prx II KO mice than that in WT mice. We then measured the bone parameters in the tibiae of the mice (Fig. 5c). The results obtained were similar to those of the femur (Fig. 5d). These results indicate that bones are better formed when Prx II is absent.

## Discussion

In this study, we first demonstrated that Prx II decreases osteoblast differentiation via PP2A Cα-mediated Smad1/5/9 dephosphorylation in C3H10T1/2 cells. Our data provide one of the molecular mechanisms by which Prx II inhibits osteoblast differentiation. Several reports have indicated that antioxidants increase osteoblast differentiation^33–35^. According to our data, one of the antioxidant enzymes Prx II suppresses BMP2-induced Smad1/5/9 phosphorylation. In addition, Prx II expression was upregulated by a high dose of LPS (1 μg/ml) but not by a low dose of LPS (0.05 μg/ml). Interestingly, a low dose of LPS induced osteogenic differentiation, which did not increase the ROS levels. The normalized ROS levels are more important than differentiation on the cell fate. We carefully suggest that the inhibition of differentiation by Prx II may alleviate the LPS-induced ROS levels in osteoblasts.

Prx II is known to have many beneficial effects based on its ability to lower the intracellular ROS levels. Prx II has been shown to prevent cell senescence induced by oxidative stress. ROS accumulation induces oxidative stress, which increases cell senescence^36^. The expression of p16, an indicator of aging, is higher in Prx II-deficient MEFs than the expression in normal MEFs. In addition, the activities of extracellular-signal-regulated kinase (ERK) and p38 were higher and cell senescence progressed further^37^. Prx II also plays an important role in maintaining the lifespan of red blood cells^38^. In addition, Prx II has been reported to be involved in the production of red blood cells by protecting DNA from damage, thereby maintaining normal erythropoiesis^39^. Prx II also binds to hemoglobin and plays an important role in preventing hemolytic anemia caused by oxidative stress^40^. However, there have also been studies demonstrating the negative effects of Prx II. Prx II increases the development, metastasis, and invasion of a variety of cancers (colon cancer, breast cancer, and pancreatic cancer) because of its ability to reduce oxidative stress^41–44^. So far, no study has been conducted on whether Prx II has a positive or negative effect on osteoblast differentiation. Therefore, we focused on how Prx II affects osteoblast differentiation.

The level of intracellular ROS affects cell survival, and apoptosis is induced at high ROS levels^45^. In vivo, osteoblast cell death is common and plays an important role in regulating bone metabolism. Apoptosis has been reported to be involved in mineralization and to be capable of increasing mineralization in the extracellular matrix^46–48^. To reduce the production of ROS, the cells were cultured in 2% oxygen; the expression levels of SOD1, 2, and CAT were decreased and early-stage osteoblast differentiation was inhibited^49^. It is particularly important to maintain a certain level of ROS during osteoblast differentiation.

Osteoblasts express various genes such as Id1, Dlx5, and Runx2, during differentiation^16,17,50^. BMPs induce osteoblast differentiation through Smad signaling^20,23^. We found that Prx II negatively regulates osteoblast differentiation in C3H10T1/2 cells. To elucidate the mechanism by which Prx II regulates osteoblast differentiation, we evaluated the effects of treatment with LPS and BMP2 by overexpressing Prx II in a Prx II KO cell line.

The regulation of protein phosphorylation and dephosphorylation is important to process cell growth and differentiation by activating proteins. The overexpression of Prx II decreased Smad1/5/9 phosphorylation and it caused an increase in the expression of PP2A Cα; however, LPS treatment did not induce the expression of PP2A Cα in Prx II KO cells. PP2A Cα has been reported to inhibit osteoblast differentiation and bone formation, and its expression has been shown to increase under excessive oxidative stress in osteoblasts^51–53^. PP2A Cα is also involved in the differentiation of osteoclasts^54^. Prx II is an antioxidant enzyme and has been shown to reduce ROS in mesenchymal stem cells. Further study is needed to determine how Prx II regulates the expression of PP2A Cα.

In summary, this study demonstrates that the LPS-stimulated induction of Prx II expression decreased osteoblast differentiation via PP2A Cα expression in C3H10T1/2 cells. We propose that Prx II-induced PP2A Cα inhibits BMP2-induced osteoblast differentiation by increasing Smad1/5/9 dephosphorylation.
